# Push-Pull Effects of Three Plant Secondary Metabolites on Oviposition of the Potato Tuber Moth, *Phthorimaea operculella*

**DOI:** 10.1673/031.013.12801

**Published:** 2013-11-21

**Authors:** Y.F. Ma, C. Xiao

**Affiliations:** 1 Plant Protection College, Yunnan Agricultural University, Kunming 650201 P. R. China; 2 Current address: Dehong Teacher's College, Dehong, Yunnan Province 678400 P. R. China

**Keywords:** azadirachtin, eucalyptol, heptanal

## Abstract

The push-pull effects of three plant secondary metabolites, azadirachtin, eucalyptol, and heptanal, on the oviposition choices of potato tubers by the potato tuber moth, *Phthorimaea operculella* (Zeller) (Lepidoptera: Gelechiidae) were tested in the laboratory. Azadirachtin at concentrations from 1.5 to 12 mg/L had a significant repellent effect on oviposition. Eucalyptol at concentrations from 3 to 12 mg/L promoted oviposition. Heptanal promoted oviposition at low concentrations from 0.1875 to 3.0 mg/L but repelled it at higher concentrations from 12 to 24 mg/L. The combination of azadirachtin (12 mg/L) with eucalyptol (3.0 mg/L) resulted in a significant pushpull effect of 56.3% on oviposition. The average maximum push-pull effects occurred with the combinations of azadirachtin with heptanal (12 and 0.375 mg/L, respectively; 38.7% push-pull effect), heptanal with eucalyptol (12 and 6 mg/L, respectively; 31.4% push-pull effect), and heptanal (high concentration) with heptanal (low concentration) (12.0 and 0.375 mg/L, respectively; 25% push-pull effect).

## Introduction

Plant secondary metabolites are important for herbivore insects to differentiate hosts from non-hosts (Dethier 1982; Bruce et al. 2005). Non-host plants generally produce chemicals that deter herbivore egg laying (Unnithan et al. 1990; Dimock et al. 1991). Conversely, the host plant volatiles can attract female herbivores to deposit eggs (Dethier 1982; Bruce et al. 2005). Therefore, combining host and nonhost chemicals could be used to manipulate the pests' host choice (Unnithan et al. 1990; Dimock et al. 1991; Foster et al. 1997). The use of combinations of behavior-modifying chemical stimuli to manipulate the distribution and abundance of pests on crops has been called the push-pull strategy and used to reduce crop damage (Shelton et al. 2006; Cook et al. 2007).

The potato tuber moth, *Phthorimaea operculella* (Lepidoptera: Gelechiidae), is one of the main potato pests. The females usually deposit single or groups of eggs on the undersides of the leaflets and on tubers in storage. They lay the eggs near the eyes and surface scars. The newly emerged larvae are found on foliage, on stems, and in the tubers of the potatoes. The damage is most severe on stored tubers. The larvae develop in the shallow and deep portions of the tuber tunnels, which became packed with excrement. Infested tubers finally become rotten and lose edible value (Foot 1979; Gamboa et al. 1990).

Azadirachtin is the main ingredient of neem, *Azadirachta indica* Jusss (Sapindales: Meliaceae), seed oil (Naumann et al. 1995). It exhibits significant anti-feeding and oviposition- deterring effects against many insect pests such as moths, beetles, plant hoppers, and aphids (Mordue et al. 1996; Cherry et al. 2010). Preliminary results also showed that azadirachtin deters oviposition by *P. operculella* on potato tubers (Kang et al. 2007). Eucalyptol (1, 8-cineole) and heptanal are common ingredients in essential oils of many plants (Lewis et al. 1988; Pålsson et al. 2008). Preliminary studies demonstrated that they encouraged *P. operculella* oviposition (Kang et al. 2007).

Therefore, the use of azadirachtin as a deterrent and eucalyptol or heptanal as attractants in combination may be used to manipulate *P. operculella* oviposition and reduce potato tuber damage. Here we report the push-pull effects of these three plant secondary metabolites on the oviposition behavior of potato tuber moths.

## Materials and Methods

### Insect source

Newly hatched larvae were introduced at a density of 20 larvae per tuber (130 ± 2 g). Ten infested tubers were placed in a mesh cage (L × W × H = 35 × 35 × 35 cm) in which sand was provided as a pupation medium to allow easy harvesting of pupae. They were kept under suitable environmental conditions (24 ± 2 °C, a 14:10 L:D photoperiod, and 70 ± 5% RH). After adult eclosion, 20 couples were confined in a plastic cylindrical container (13.0 cm in diameter and 14.5 cm high) for copulation and provided with 10% honey water. The open end of the container was closed with mesh on which a piece of filter paper was placed for egg deposition.

### Effects of three chemicals on oviposition tuber choices by *P. operculella*

Azadirachtin A (96%, Sigma, http://www.sigmaaldrich.com), eucalyptol (95%, Acros Organics, http://www.acros.com), heptanal (99%, TCI America, http://www.tcichemicals.com), and ethanol (>99.5%) were used. Azadirachtin was dissolved in ethanol (99%) (v/v = 1:2) and diluted with distilled water to achieve a series of dilutions (12.0 mg/L, 6.0 mg/L, 3.0 mg/L, 1.5 mg/L, and 0.75 mg/L). Tween-80 (> 99%, Sigma-Adrich, http://www.sigmaaldrich.com) was added to each solution at a final concentration of 1.0 mg/L. Eucalyptol solutions (12.0 mg/L, 6.0 mg/L, 3.0 mg/L, 1.5 mg/L, and 0.75 mg/L) and heptanal solutions (24.0 mg/L, 12.0 mg/L, 6.0 mg/L, 3.0 mg/L, 1.5 mg/L, 0.75 mg/L, and 0.375 mg/L) were prepared in the same manner. Control solutions consisting of the same amounts of ethanol, Tween-80, and distilled water were prepared as described above. Each solution was prepared one hour before the experiment.

**Table 1.**
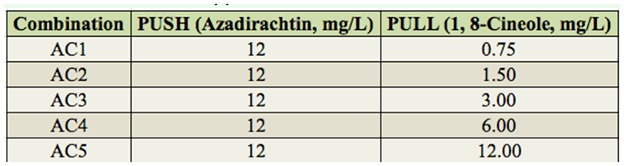
Concentrations used to test the combination of azadirachtin and eucalyptol.

**Table 2.**
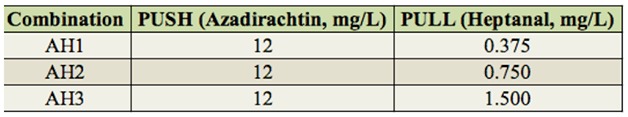
Concentrations used to test the combination of azadirachtin and heptanal.

**Table 3.**
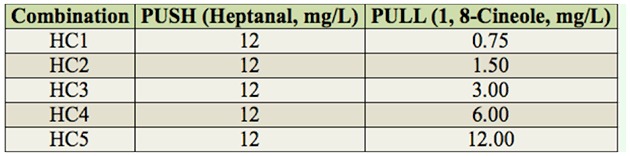
Concentrations used to test the combination of heptanal and eucalyptol.

**Table 4.**
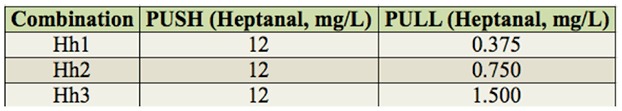
Concentrations used to test the combination of different concentrations of heptanal.

For testing the effects of single chemicals on oviposition, three treated potato tubers (130 ± 2 g) and three control tubers were immersed in the chemical solutions for 5 seconds and then air-dried for 1 hour. The experimental and control tubers were then placed 15 cm apart in a mesh cage. Twenty couples of freshly emerged adult *P. operculella* were placed in the cage and supplied with 10% honey water. The cages were kept under the same environmental conditions as above. The eggs on the surfaces of the potato tubers were counted and recorded on the third day. Each test was repeated 4–5 times.

The one-to-one combinations of chemicals tested were azadirachtin (push) with eucalyptol (pull) (Table 1), azadirachtin (push) with heptanal (pull) (Table 2), heptanal (high concentration, push) with eucalyptol (pull) (Table 3), and heptanal (high concentration, push) with heptanal (low concentration, pull) (Table 4). All tests were performed as above with 3–5 replicates.


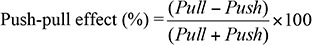


“Pull” indicates the egg number on the tubers treated with the attractant. “Push” indicates the egg number on the tubers treated with the repellent.

### Statistical analysis

The comparisons between the treatment and control groups were analyzed using a Chisquare test at the level of 0.05 (MS Excel 2007). For comparisons of push-pull effects between combinations, an analysis of variance (ANOVA) and a Duncan's Multiple range test were used at the level of 0.05 (SPSS 13.0).

## Results

### Effects of single chemicals on the oviposition choices of *P. operculella* females

**Figure 1.**
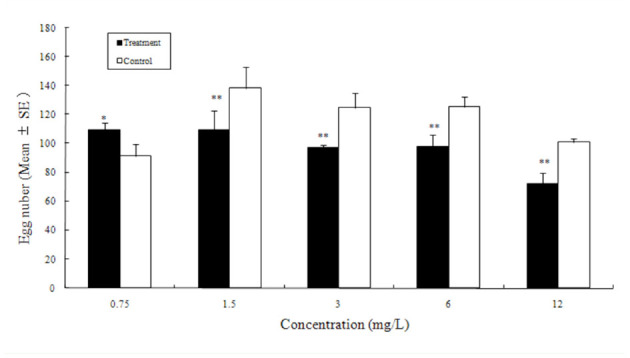
Effect of azadirachtin on potato tuber oviposition choices by *Phthorimaea operculella*. The single and double asterisks indicate significant differences from the control at the levels of 0.05 and 0.01, respectively (Chi-square test, *n* = 4). High quality figures are available online.

Azadirachtin significantly deterred oviposition by *P. operculella* females at concentrations ranging from 1.5 mg/L to 12 mg/L but significantly promoted oviposition at 0.75 mg/L (Figure 1).

**Figure 2.**
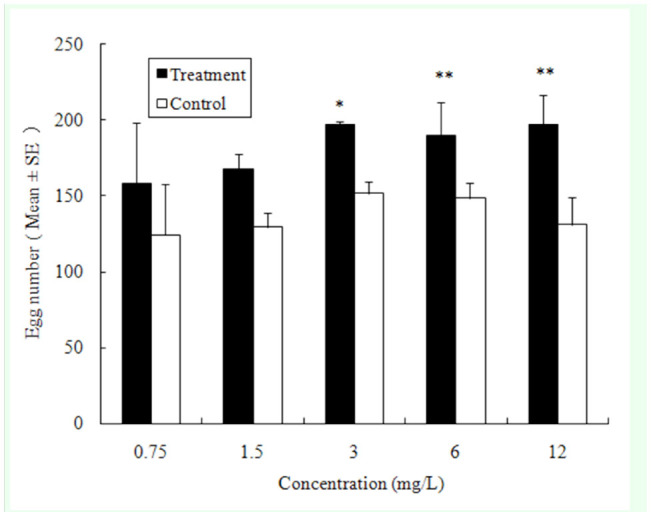
Effect of eucalyptol on potato tuber oviposition choices by *Phthorimaea operculella*. The single and double asterisks indicate significant differences from the control at the levels of 0.05 and 0.01, respectively (Chi-square test, *n* = 5). High quality figures are available online.

**Figure 3.**
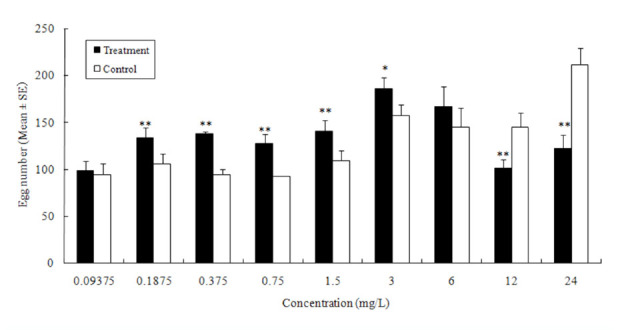
Effect of heptanal on potato tuber oviposition choices by *Phthorimaea operculella*. The single and double asterisks indicate significant differences from the control at the levels of 0.05 and 0.01, respectively (Chi-square test, *n* = 4). High quality figures are available online.

The eucalyptol solutions above 0.75 mg/L resulted in significant oviposition promotion (Figure 2). The heptanal solutions at low concentrations ranging from 0.375 mg/L to 1.50 mg/L also resulted in significant oviposition promotion, but those at concentrations above 12.00 mg/L inhibited oviposition (Figure 3).

### Push-pull effects one one-to-one combinations of the three chemicals on *P. operculella* oviposition

**Figure 4.**
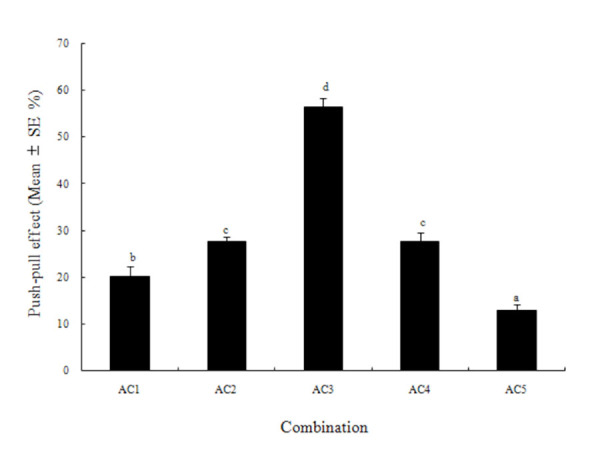
Push-pull effect of the combination of azadirachtin and eucalyptol on the potato tuber oviposition choices by *Phthorimaea operculella*. Different letters above the error bars indicate significant differences between the combinations at the level of 0.05 (*n* = 4; homogeneity of variance: df1 = 4, df2 = 15, *p* = 0.667; ANOVA: *F* = 81.42, *p* = 0.0004). High quality figures are available online.

In all of the combinations of azadirachtin (push) and eucalyptol (pull), more eggs were deposited on the potato tubers containing eucalyptol than on those with azadirachtin. In particular, the AC3 (Table 1) combination of azadirachtin (12.00 mg/L) and eucalyptol (3.00 mg/L) resulted in the strongest Push-pull effect, which on average reached 56.3% (Figure 4).

**Figure 5.**
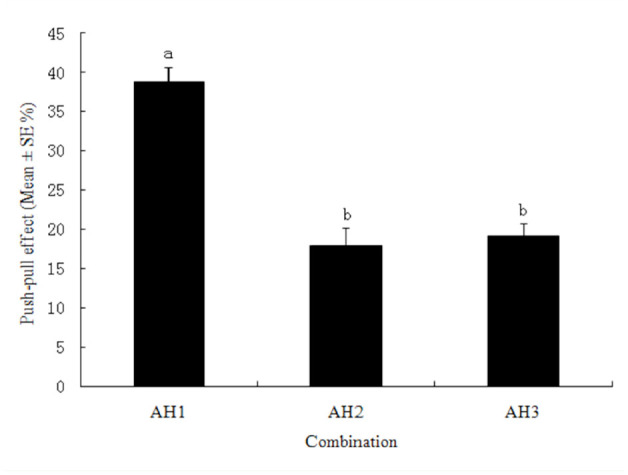
Push-pull effect of azadirachtin and heptanal (low concentration) on the potato tuber oviposition choices by *Phthorimaea operculella*. Different letters above the error bars indicate significant differences between the combinations at the level of 0.05 (*n* = 3; homogeneity of variance: df1 = 2, df2 = 6, *p* = 0.788; ANOVA: *F* = 34.695, *p* = 0.001). High quality figures are available online.

In the AH1 (Table 2) combination of azadirachtin (12.00 mg/L, push) and heptanal (0.375 mg/L, pull), the push-pull effect reached 38.7%, which was significantly greater than the effects of the combinations between azadirachtin and higher concentrations of heptanal (Figure 5).

**Figure 6.**
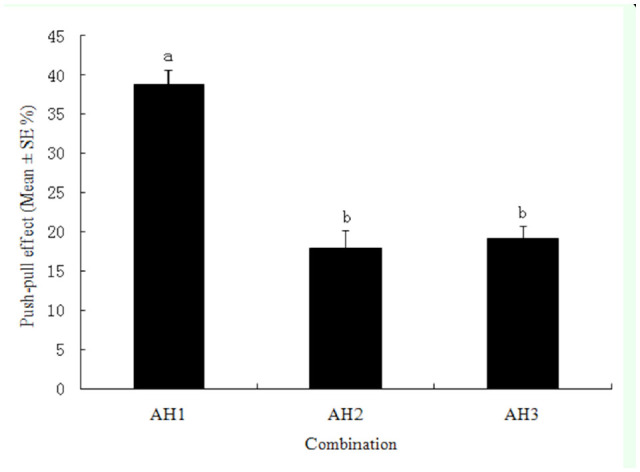
Push-pull effect of the combination of heptanal (high concentration) and 1, 8-cineole on the potato tuber oviposition choices by *Phthorimaea operculella*. Different letters above the error bars indicate significant differences between combinations at the level of 0.05 (*n* = 5; homogeneity of variance: df1 = 4, df2 = 15, *p* = 0.37; ANOVA: *F* = 3.684, *p* = 0.028). High quality figures are available online.

Push-pull effects ranged from 19% to 31% in all of the combinations of heptanal (high conconcentration, Push) and eucalyptol (Pull). The push-pull peak appeared in the HC4 (Table 3) combination (12 mg/L heptanal and 6 mg/L eucalyptol) with an average push-pull effect of 31.4%. There were no significant differences in the push-pull effects of the combinations of heptanal with other concentrations of eucalyptol (Figure 6).

**Figure 7.**
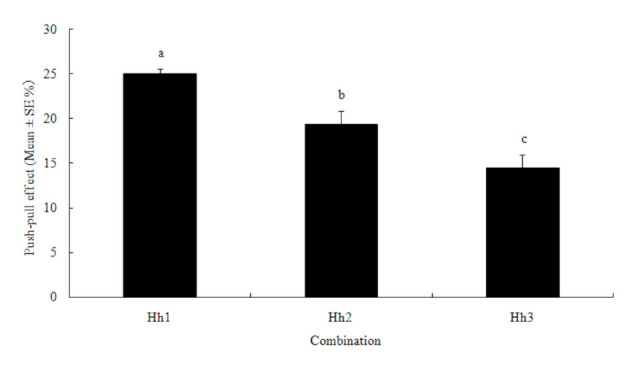
Push-pull effect of the combination of different concentrations of heptanal (push and pull) on potato tuber oviposition choices by *Phthorimaea operculella*. Different letters above the error bars indicate significant differences between the combinations at the level of 0.05 (*n* = 3; homogeneity of variance: df1 = 4, df2 = 15, *p* = 0.37; ANOVA: *F* = 3.684, *p* = 0.028). High quality figures are available online.

Among the combinations of heptanal (high concentration, push) with heptanal (low concentration, pull), Hh1 (Table 4) (12 mg/L and 0.375 mg/L heptanal) showed a significant push-pull effect with an average of 25%, which was significantly greater than the effects of Hh2 and Hh3 (Figure 7).

## Discussion

Potato tubers in storage were seriously damaged by *P. operculella*. Tuber infestation was highest in Kenya, with 21.3% of tubers being damaged (Keller 2002). More seriously, 100%of the tubers were damaged when they were stored for more than four months in some villages of the Yunnan Province (Kang et al. 2007). Therefore, it is necessary to develop new techniques to reduce the tuber damage by *P. operculella*.

Our results showed that it is practical to use the push-pull strategy to manipulate *P. operculella* oviposition on potato tubers. The pushpull effect reached 56.3% when azadirachtin (12.0 mg/L) as the push factor and eucalyptol (3.0 mg/L) as the pull factor were used in combination (Figure 4). Nearly 80% of the eggs on average were laid on the potato tubers treated with eucalyptol. Therefore, azadirachtin could be used to protect tubers from infestation by potato tuber moths, and tubers sprayed with suitable amounts of eucalyptol might be used to trap as many eggs as possible.

Heptanal had dual bioactivities against oviposition: attraction at low concentrations (below 3.0 mg/L) and repellency at high concentrations (above 12 mg/L) (Figure 3). This showed the potential value of heptanal in a push-pull strategy. Our experimental results showed that the push-pull effects with heptanal combinations mainly depended on the concentration gaps between the factor (high concentration) and pull factor (low concentration), although the maximum effect of the combination only reached 31.4 % (Fig. 7). This result showed that a perfect push-pull effect might not be gained through heptanal concentration combinations.

The experimental results also showed that azadirachtin at quite low concentration (0.75 mg/L) resulted in significant oviposition attraction (Figure 1). We plan to further test *P. operculella* attraction to azadirachtin.

## References

[bibr01] Bruce TJ, Wadhams LJ, Woodcock CM. (2005). Insect host location: a volatile situation.. *Trends in Plant Science*.

[bibr02] Cherry R, Nuessly G. (2010). Repellency of the biopesticide, azadirachtin, to wireworms (Coleoptera: Elateridae).. *Florida Entomologist*.

[bibr03] Cook SM, Khan ZR, Pickett JA. (2007). The use of Push-pull strategies in integrated pest management.. *Annual Review of Entomology*.

[bibr04] Dethier VG. (1982). Mechanism of host plant recognition.. *Entomologia Experimentalis et Applicata*.

[bibr05] Dimock MB, Renwick JAA. (1991). Oviposition by field populations of *Pieris rapae* (Lepidoptera: Pieridae) deterred by an extract of a wild crucifer.. *Environmental Entomology*.

[bibr06] Foot M. (1979). Bionomics of the potato tuber moth, *Phthorimaea operculella* (Gelechiidae), at Pukekohe.. *New Zealand Journal of Zoology*.

[bibr07] Foster SP, Harris MO. (1997). Behavioral manipulation methods for insect pest management.. *Annual Review of Entomology*.

[bibr08] Gamboa M, Notz A. (1990). Biología de *Phthorimaea operculella* (Zeller) (Lepidoptera: Gelechiidae) en papa (Solanum tuberosum).. *Revista de la Facultad de Agronomia* (Maracay).

[bibr09] Kang M, Tan ZX, Ren JT, Su PJ, Xiao C. (2007). Effect of azadirachtin and cineole on oviposition by *Pththoimaea operculella* females.. *Anhui Agricultural Science Bulletin*.

[bibr10] Keller S., Kroschel J (2002). Integrated pest management of the potato tuber moth in cropping systems of different agro-ecological zones.. *Advances in crop research*..

[bibr11] Lewis JA, Moore CJ, Fletcher MT, Drew RA, Kitching W. (1988). Volatile compounds from the flowers of *Spathiphyllum cannaefolium*.. *Phytochemistry*.

[bibr12] Mordue (Luntz) AJ, Nisbet AJ, Nasiruddin M, Walker E. (1996). Differential thresholds of azadirachtin for feeding deterrence and toxicity in locusts and an aphid.. *Entomologia Experimentalis et Applicata*.

[bibr13] Naumann K, Isman MB. (1995). Evaluation of neem *Azadirachta indica* seed extracts and oils as oviposition deterrents to noctuid moths.. Entomologia *Experimentalis et Applicata*.

[bibr14] Pålsson K, Jaenson TGG, Baeckström P, Borg-Karlson A-K. (2008). Tick repellent substances in the essential oil of *Tanacetum vulgare*.. *Journal of Medical Entomology*.

[bibr15] Shelton AM, Badenes-Perez FR. (2006). Concepts and applications of trap cropping in pest management.. *Annual Review of Entomology*.

[bibr16] Unnithan GC, Saxena KN. (1990). Diversion of oviposition by *Atherigona soccata* (Diptera: Muscidae) to nonhost maize with sorghum seedling extract.. *Environmental Entomology*.

